# Visualization and Quantification of Transmembrane Ion Transport into Giant Unilamellar Vesicles**

**DOI:** 10.1002/anie.201410200

**Published:** 2014-12-30

**Authors:** Hennie Valkenier, Néstor López Mora, Alexander Kros, Anthony P Davis

**Affiliations:** School of Chemistry, University of BristolCantock's Close, Bristol BS8 1TS (UK); Leiden Institute of Chemistry, Leiden University2300 RA Leiden (The Netherlands)

**Keywords:** anions, giant unilamellar vesicles, ion transport, membranes, supramolecular chemistry

## Abstract

Transmembrane ion transporters (ionophores) are widely investigated as supramolecular agents with potential for biological activity. Tests are usually performed in synthetic membranes that are assembled into large unilamellar vesicles (LUVs). However transport must be followed through bulk properties of the vesicle suspension, because LUVs are too small for individual study. An alternative approach is described whereby ion transport can be revealed and quantified through direct observation. The method employs giant unilamellar vesicles (GUVs), which are 20–60 μm in diameter and readily imaged by light microscopy. This allows characterization of individual GUVs containing transporter molecules, followed by studies of transport through fluorescence emission from encapsulated indicators. The method provides new levels of certainty and relevance, given that the GUVs are similar in size to living cells. It has been demonstrated using a highly active anion carrier, and should aid the development of compounds for treating channelopathies such as cystic fibrosis.

Transmembrane ion transport is a key process in biology. While membranes are intrinsically impermeable to ions, the cell needs to ingest and excrete charged species to sustain metabolism, avoid osmolysis, and perform specialist functions. Biological ion transport is mediated by proteins,[1] but it has long been known that small molecules can have similar effects. The naturally derived ionophore antibiotics act by promoting cation transport across cell membranes,[2] and synthetic analogues can also be effective. More recently it has been shown that anion transport is also achievable by synthetic systems,[3] including both channels[4] and carriers.[5] In this case there is particular interest in replacing the activity of defective natural systems, yielding potential therapies for “channelopathies” such as cystic fibrosis (CF). Research on both cation and anion transport is ongoing, attracting a substantial community of bioorganic and supramolecular chemists.[6]

The study of ion transport by small molecules is commonly performed using large unilamellar vesicles (LUVs),[7] which are spherical assemblies of lipids circa 100–200 nm in diameter in which the membrane isolates a small volume of interior aqueous solution. Transport into or out of the vesicles can then be studied by techniques such as fluorescence (using ion-sensitive fluorophores), NMR spectroscopy (using shift reagents to distinguish between interior and exterior), or ion-selective electrodes. These methods are easy to implement but have certain disadvantages, especially for quantitative transport studies. Many of the problems relate to their small size, which is less than the wavelength of visible light and hampers imaging by light microscopy.[8] For example, while the standard method of production (extrusion through a microporous filter) allows control over size, a range of diameters are always present in a given sample. Secondly, even though unilamellar vesicles are thermodynamically favored, the self-assembly of lipids can also produce multilamellar vesicles or other structures. Their presence may be inferred from the bulk behavior of the suspension, but cannot be observed directly. Thirdly, a typical experiment on the LUV suspension will involve addition of a transporter or substrate, then the observation of a change (for example in bulk fluorescence), which reports transport into or out of vesicles. In principle the same change can often be produced by vesicle bursting rather than transport. While circumstances and controls may suggest that bursting is unlikely, doubts may persist. Fourthly, the LUVs are about two orders of magnitude smaller than most cells, and this affects their value as cell models. In particular, the LUVs possess much higher surface-to-volume ratios, which increases their sensitivity to transport processes (see discussion below). Agents that cause major changes in LUVs may thus have limited potential for biological activity.

Herein we report a new method for studying ion transport that circumvents the above problems and allows direct, unambiguous observation of the transport process. Instead of LUVs, the method employs individual giant unilamellar vesicles (GUVs)[9] with diameters of 20–60 μm, similar to many cells and readily observable by microscopy. Fluorescence microscopy of GUVs has been used to study passive diffusion of peptides[10] and organic compounds[11] through membranes, passive diffusion of dyes through pores formed by peptides[12] or proteins[13] and to qualitatively study the presence and performance of membrane proteins.[14] However, as far as we know, this is the first report in which the technique has been applied to the transport of inorganic ions. The method has been used to visualize and quantify chloride transport by a powerful anion transporter, and could in principle be applied to many other ion transport processes.

The new method as applied herein is illustrated in Figure 1. The transport process is Cl^−^/NO_3_^−^ exchange by the bis(thioureido)decalin **1**, an anion carrier (anionophore) that has recently been prepared by the Bristol group.[15] Giant vesicles are formed in which the transporter is located in the bilayer membrane and the chloride-sensitive fluorophore lucigenin **2** is trapped in the aqueous interior. Both interior and exterior aqueous phases contain NaNO_3_ (225 mm). When NaCl is added to the exterior solution, the chloride is carried through the membrane by **1** and makes contact with lucigenin, quenching fluorescence.[16,17] Counter-transport of nitrate maintains electroneutrality.

**Figure 1:**
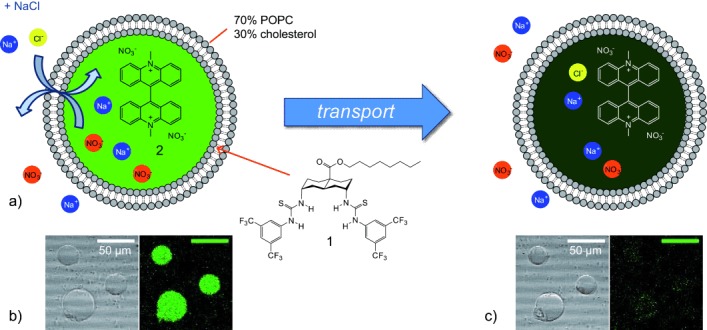
a) Representation of the transport of chloride by transporter 1 into giant unilamellar vesicles (GUVs). Upon addition of a solution of NaCl, the transporter exchanges exterior chloride for interior nitrate. The chloride that is transported into the GUVs quenches the fluorescence of lucigenin 2 present in the interior of the vesicles. b,c) Bright-field (left) and confocal fluorescence microscopy images (right) show three giant vesicles with transporter 1 (0.1 mol % of total lipid) preincorporated in the membrane before (b) and after (c) addition of NaCl.

The transfer of this assay to GUVs required a powerful transporter. Absolute transport rates into vesicles depend on the surface area, while the rate of change of substrate concentration depends on the interior volume. For a given vesicle composition, d[substrate]/d*t* scales with the surface-to-volume ratio, which decreases linearly with vesicle diameter.[18] Concentration changes for a 20 μm GUV should therefore be 100 times slower than for a 200 nm LUV, and only the more active transporters are likely to give observable effects. Anionophore **1** features strongly anion-binding thioureido groups preorganized on a *trans*-decalin scaffold,[19] and a uniformly lipophilic exterior which appears to favor passage through bilayer membranes. It had shown exceptional activity in conventional LUV-based transport experiments[15] and was therefore most likely to succeed in the new test system.

Also needed was a method for preparing GUVs of well-defined size from lipids doped with transporter, at high ionic strength and with a cholesterol rich lipid mixture. The problem was solved using a technique developed by the Leiden group, in which the GUVs are grown on a cross-linked dextran–(polyethylene glycol) hydrogel substrate.[20] Adjusting the density of cross-links allows control of vesicle size between about 20 and about 100 μm. In the present case GUVs were grown from 1-palmitoyl-2-oleoylphosphatidylcholine (POPC) and cholesterol (7:3 ratio), plus a varied amount of transporter **1** (0 mol %, 0.01 mol %, 0.04 mol %, and 0.1 mol % of total lipid), using a hydrogel designed to yield vesicles of 10–40 μm diameter.[21] The hydrogel with the lipid film containing the transporter was rehydrated with a solution of 225 mm NaNO_3_, 0.8 mm lucigenin and 200 mm sucrose. The resulting giant vesicles were transferred into a microscopy chamber and the external solution was replaced by perfusion with a solution of 225 mm NaNO_3_ and 200 mm glucose. This procedure removed the external lucigenin while lowering the density of the medium, causing the GUVs to settle on the viewing surface. The giant vesicles were imaged both in bright-field mode and when excited with a 488 nm laser in a confocal fluorescence microscope. After 30–60 s, 25 μL 1 m NaCl solution was added to the microscopy chamber with a microsyringe, giving rise to an external chloride concentration of about 50 mm. As expected, the intensity of lucigenin emission was observed to decay significantly over a period of about 5 min (Figure 2). Bright-field images confirmed the presence of intact GUVs after quenching (see Figure 1 b,c and the Supporting Information), showing that the apparent disappearance of vesicles was not due to bursting. No fluorescence decay was observed in the absence of transporter (Figure 2 a,e), and the rate of decay was clearly dependent on the amount of transporter **1** added (Figure 2 b–d,f–h). Time-lapse videos from the fluorescence microscope are provided as Supporting Information (.avi files SV1–4). The possibility of dye leakage was ruled out by a control experiment in which lucigenin was replaced carboxyfluorescein. In this case, fluorescence emission from the vesicles underwent negligible change (Supporting Information, Figure S15). Our studies thus provide unambiguous confirmation that bis(thiourea) **1** does indeed promote chloride transport across bilayer membranes, while preserving the lipid membrane and GUV integrity.

**Figure 2:**
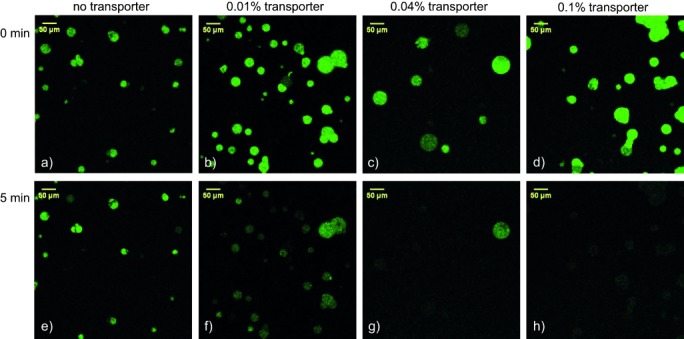
Confocal fluorescence microscopy images of lucigenin-containing GUVs, incorporating varying amounts of transporter 1, before (a–d) and about 5 min after addition of NaCl (e–h).

To obtain more insight into the process, we quantified the fluorescence intensity of the giant vesicles within each frame of the recorded time lapses. We then normalized and averaged the fluorescence intensities of the vesicles within one chamber (and from one NaCl addition experiment) to obtain curves that show the average fluorescence over time (Figure 3). The experiment was performed twice for most concentrations and four times with the GUVs containing 0.01 % transporter. Figure 3 clearly shows how quenching and thus anion transport is fastest when 0.1 % transporter is present (red) and how it is only slightly slower when 0.04 % transporter is present (blue). When only 0.01 % transporter is present (green), the fluorescence intensity has not yet plateaued within 250 s and transport is still ongoing. For this reason, the fluorescence intensities of the GUVs with 0.01 % transporter were monitored over 20 min (see Supporting Information for the full data sets). We also monitored the fluorescence intensity of GUVs over time without adding NaCl to test for photobleaching of lucigenin. As indicated by the black line in Figure 3,[22] no photobleaching was observed in the first 5 min and even after 20 min of monitoring the bleaching was still below 7 %, which is insignificant compared to the loss of fluorescence by quenching caused by transport of chloride.

**Figure 3:**
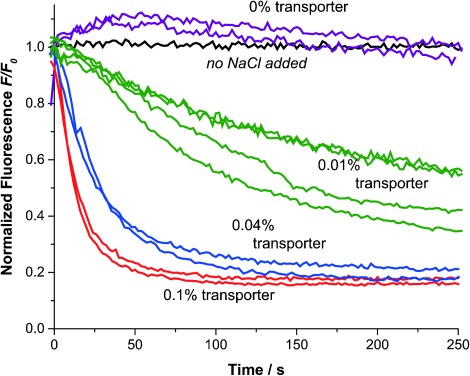
Average traces of the normalized lucigenin emission intensity after addition of 50 mm NaCl to GUVs without transporter (purple), with 0.01 % transporter (green), with 0.04 % transporter (blue), and with 0.1 % transporter present (red) or without NaCl added (black).

Careful examination of all the fluorescence decay profiles reveals that while most GUVs within one experiment have similarly shaped fluorescence vs. time curves (forming a distribution that is due to the variation in sizes of the vesicles), certain vesicles show distinct behavior (see full datasets in the Supporting Information). For instance, the fluorescence of one giant vesicle in Figure 2 g remains visible where all the others have disappeared into the background (these distinctively slower traces have not been included in the average curves in Figure 3). Suspecting that this distinct transport behavior was due to multilamellarity of certain vesicles, we repeated the experiments employing 0.01 % and 0.04 % transporter with lissamine rhodamine B-labeled lipid added to the membrane (0.1 mol %; Supporting Information, videos SV5 and SV6). This allowed us to visualize the membranes of the vesicles upon excitation with a 532 nm laser. Multilamellar membranes give higher intensities of rhodamine fluorescence compared to unilamellar membranes.[23] The results of one of these experiments employing 0.01 % transporter **1** are presented in Figure 4. For typical GUVs, the intensity of red fluorescence emitted from the membranes is about 25 units. However, for the vesicles labeled C and D in Figure 4 a the observed intensity is double this value (ca. 50 units) while the intensities from the membranes of GUVs A and B reach 200–250 and 100–150 units, respectively (for details, see the Supporting Information, Figure S12). In Figure 4 b we clearly see that the vesicles labeled A–D display a stronger intensity of fluorescence of lucigenin, even after 20 min. This is also seen in the normalized fluorescence traces of the individual vesicles as plotted in Figure 4 c. After 1000 s the fluorescence intensity of most vesicles has reached the plateau value of about 20 % of the initial fluorescence, while plots from vesicles A–D show much slower decays. As giant vesicles C and D show a rhodamine emission intensity which is double the value of the majority of the vesicles, and both have identical curves, these are likely to have a double lipid bilayer. Vesicles A and B, with even stronger rhodamine emission and slower lucigenin quenching, are likely to have higher orders of multilamellarity. Vesicles A–D are therefore excluded from further quantitative analysis.

**Figure 4:**
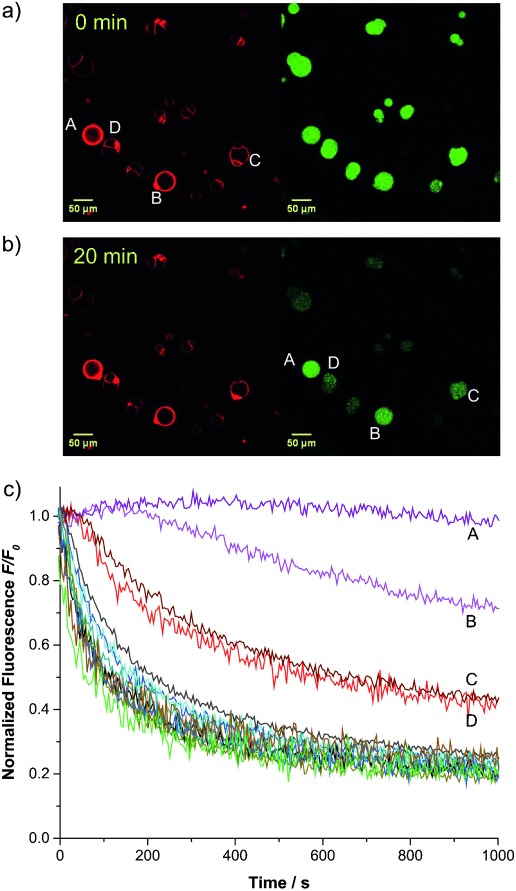
a) Fluorescence microscopy images of GUVs containing lucigenin and incorporating rhodamine-labeled lipid +0.01 % transporter 1. Left: Illumination at 532 nm visualizes the rhodamine in the bilayer. Right: Illumination at 488 nm excites the lucigenin. b) As above, 20 min after addition of NaCl. c) Traces of the lucigenin fluorescence intensity of individual vesicles over time after addition of 50 mm NaCl. The four vesicles that display stronger rhodamine fluorescence, and their corresponding traces, are labeled A–D.

Having found a method to distinguish unilamellar and multilamellar giant vesicles, we were able to quantify transport into the unilamellar vesicles. We focused on the slower-transporting GUVs containing 0.01 % bis(thiourea) **1** to minimize errors that are due to the addition of NaCl at the start of the experiment. Relatively slow and careful addition is necessary to avoid disturbing the GUVs in the field of the microscope. The fluorescence decay data were analyzed using a procedure previously employed for **1** in LUVs.[15] Values for *F*_0_/*F*[24] were fitted to a single exponential decay function, which was converted to chloride concentrations by assuming a limiting intravesicular chloride concentration of 50 mm. This was then used to calculate an initial rate of chloride transport per transporter molecule, taking account of the size of the vesicle. The analysis was performed for 56 GUVs from 6 experiments (all with 0.01 % transporter), giving an average initial rate per transporter of 820±260 Cl^−^ s^−1^. This value is similar to that obtained from experiments on bulk LUV solutions using the same transporter (850 Cl^−^ s^−1^).[15] However, because the present work was performed on vesicles of known diameters and lamellarities, characterized by microscopy, we believe it is much more reliable. For a full description of the analysis procedure, see the Supporting Information.

In conclusion, we have devised a new method whereby ion transport by small molecules into individual giant unilamellar vesicles can be observed and quantified. By directly visualizing transport into GUVs, the approach offers a high level of certainty and integrity compared to experiments on bulk suspensions of smaller vesicles. Instead of quantifying transport into a population of vesicles with a distribution of sizes, we can now analyze the transport into individual GUVs of which we can verify the lamellarity and measure the size. The method is complementary to studies in LUVs, in that it is better suited to very powerful transporters which can produce measurable effects despite the low surface-to-volume ratio of GUVs. Indeed, positive results in this test provide clear encouragement that a transporter has potential for biological activity.

In this initial demonstration the method has been used to study chloride/nitrate exchange by an anion carrier. However, it is reasonable to suppose that other types of ion transport could be investigated similarly. A number of fluorescence-based methods have been developed for following transport into LUVs.[7] For example, the pH-sensitive probe 8-hydroxy-1,3,6-pyrenetrisulfonate (HPTS) is used as a general indicator of ion transport, while other dyes have been used to follow transport of specific metal cations. Transfer of these assays to GUVs should be straightforward, while further indicators are readily available[25] and remain to be exploited.

Finally, the success of **1** at mediating Cl^−^/NO_3_^−^ exchange in this system, even at the modest loading of 0.01 %, further highlights its exceptional activity. This molecule is relatively lipophilic so that dispersion in water and delivery to cell membranes may be challenging. However if the delivery problem can be solved, for example with membrane fusion,[26] the effectiveness of **1** in cell-sized vesicles augurs well for applications in biology and medicine.
