# Supplementary material for: Visualization and Quantification of Transmembrane Ion Transport into Giant Unilamellar Vesicles
Source: Angew Chem Int Ed Engl. 2014 Dec 30;54(7):2137–41. doi: 10.1002/anie.201410200 (PMC4506561; doi:10.1002/anie.201410200)
Supplement: Supplementary file 1 [file anie0054-2137-sd1.pdf]

Angewandte Chemie
Eine Zeitschrift der Gesellschaft Deutscher Chemiker

Supporting Information

© Wiley-VCH 2014

69451 Weinheim, Germany

Visualization and Quantification of Transmembrane Ion Transport into Giant Unilamellar Vesicles**

Hennie Valkenier, Néstor López Mora, Alexander Kros, and Anthony P. Davis**

anie_201410200_sm_miscellaneous_information.pdf
anie_201410200_sm_SV1.avi
anie_201410200_sm_SV2.avi
anie_201410200_sm_SV3.avi
anie_201410200_sm_SV4.avi
anie_201410200_sm_SV5.avi
anie_201410200_sm_SV6.avi

SUPPORTING INFORMATION

Contents: Page

1. Experimental Section S2
2. Full datasets behind the average curves presented in Figure 3 of the main paper S3
3. Cross-sections of vesicles with rhodamine-labelled membranes in Figure 4a S9
4. Fitting of the fluorescence intensities of individual GUVs S10
5. Test for leakage of a fluorescent dye from the GUVs S12

1. Experimental Section

Cholesterol, 1-palmitoyl-2-oleoyl-sn-glycero-3-phosphocholine (POPC), and bovine serum albumin (BSA) were purchased from Sigma Aldrich. 1,2-Dioleoyl-sn-glycero-3-phosphoethanolamine-N-(lissamine rhodamine B sulfonyl) (ammonium salt) (rhodamine labelled lipid) was purchased from Avanti Polar Lipids and 10,10'-dimethyl-9,9'-biacridinium nitrate (lucigenin, **2**) was purchased from Tokyo Chemical Industry UK Ltd. Chloroform was deacidified by passage through a column containing activated basic alumina before the preparation of the lipid solutions. The lucigenin (0.8 mM), $NaNO_3$ (225 mM) and NaCl (1 M) aqueous solutions were prepared with Millipore grade water. Lipid solutions of POPC and cholesterol (70:30 ratio, 14 mM) were prepared in the previously deacidified chloroform and the transporter octyl t-(2,7)-bis(3-(3,5-bis(trifluoromethyl)phenyl)thioureido)-t-8a-decahydronaphthalene-r-4a-carboxylate,[1] (**1**, 84 µM solution in methanol) was added to this lipid solution at 0.01 mol%, 0.04 mol% and 0.1 mol% (relative to total lipid). Rhodamine labelled lipid was added in 0.1 mol% when membrane imaging was required.

Giant Unilamellar Vesicles (GUVs) were grown in Dex-PEG (1:1 ratio) coated microscope glass slides substrates as described previously.[2] Lipid solution (10 µL) was deposited on a hydrogel coated glass slide, then the lipid solution was dried by evaporating the chloroform under a gentle stream of Nitrogen gas. A liquid chamber was made by placing a 15 mm (OD) glass O-Ring on top of the hydrogel and sealed with high vacuum silicon grease. The lipid film was rehydrated by adding 400 µL of an aqueous solution that contains lucigenin (0.8 mM), $NaNO_3$ (225 mM) and sucrose (200 mM), into each chamber. GUVs were grown during at least 3 hours at room temperature and subsequently the solution with free floating GUVs was transferred into an eppendorf tube containing 600 µL of $NaNO_3$ (225 mM) and glucose (200 mM) aqueous solution. The visualization chamber was pre-treated with an aqueous solution of BSA (1 mg/mL, 500 µL) for one hour. The diluted solution with GUVs was transferred into the microscopy visualization chamber (500 µL per well). The GUVs were left to sediment for at least 30 minutes and the excess of non-encapsulated lucigenin was replaced by the $NaNO_3$ and glucose solution using a peristaltic perfusion pump (Instech P720, ~ 0.4 mL/min, 30 minutes), to produce a clean background for imaging the encapsulated lucigenin dye in the GUVs.

During the imaging of the GUVs in a time lapse experiment, 25 µL 1M NaCl (in $NaNO_3$ and glucose solution) was added to the well after 30-60 seconds with a microsyringe, giving a final NaCl concentration of ~50 mM.

All imaging was performed on a Leica TCS SPE confocal microscope system. Illumination was provided by a solid state laser using the 488 nm laser line (15% laser power) for scanning lucigenin's fluorescence and 532 nm laser line (10-15% laser power) for scanning lissamine rhodamine's fluorescence. Confocal microscopy was carried out using a 20× dry objective. The analysis of the images was performed in ImageJ software, by measuring the average intensity of an area corresponding to one GUV for the series of time lapsed confocal image frames.

The osmolality of a $NaNO_3$ (225 mM) solution was determined from the freezing point depression using an Osmometer Roebling Type 13 (calibrated using 100 mOsm/kg NaCl standard solution) and found to be 378 mOsm/kg (standard PBS buffer was 310 mOsm/kg). Addition of 50 mM NaCl to 225 mM $NaNO_3$ causes the osmolality to increase to 473 mOsm/kg.

---

[1] H. Valkenier, L. W. Judd, H. Li, S. Hussain, D. N. Sheppard, A. P. Davis, *J. Am. Chem. Soc.* **2014**, *136*, 12507–12512.

[2] N. López Mora, J. S. Hansen, Y. Gao, A. A. Ronald, R. Kieltyka, N. Malmstadt, A. Kros, *Chem. Commun.* **2014**, *50*, 1953–1955.

2. Full datasets behind the average curves presented in Figure 3 of the main paper

No transporter

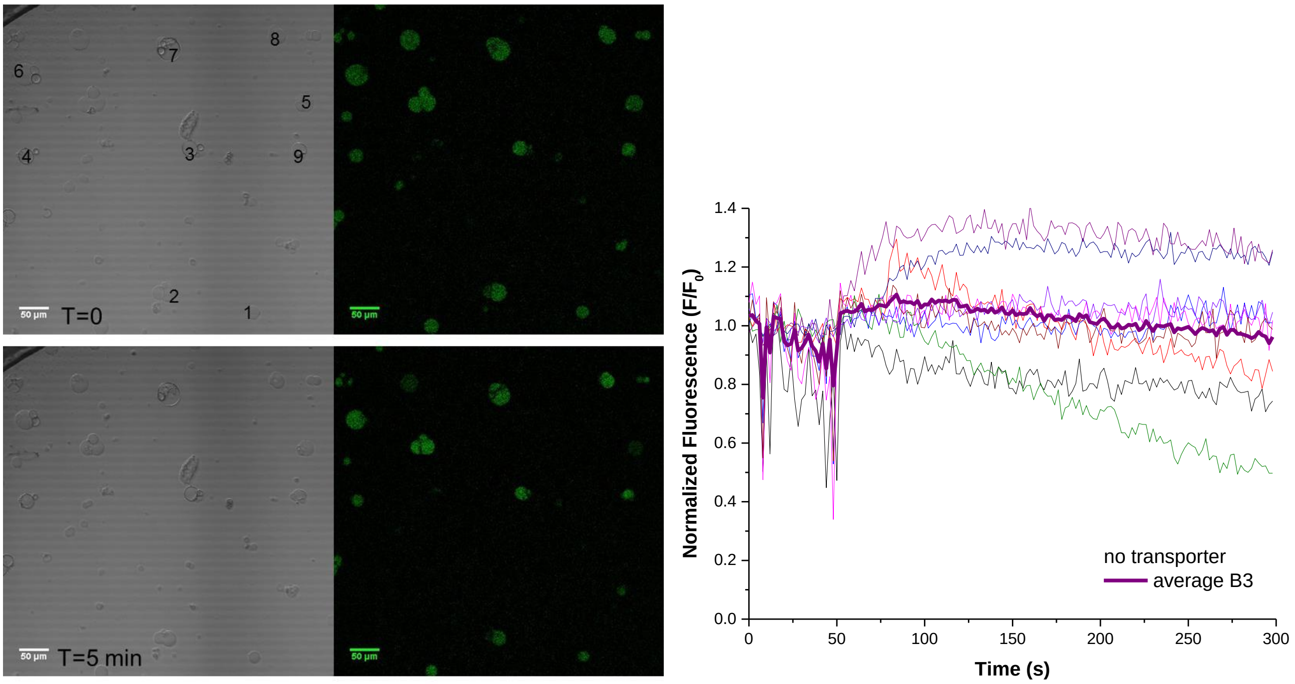

Figure S1 Bright-field and fluorescence microscopy images before (top) and after (bottom) addition of NaCl to GUVs without transporter (*experiment B3*). The normalized fluorescence intensity over time is given for the individual numbered GUVs and for the average of all numbered GUVs (fat purple line). The average diameter of the GUVs used in the average trace for 36 µm.

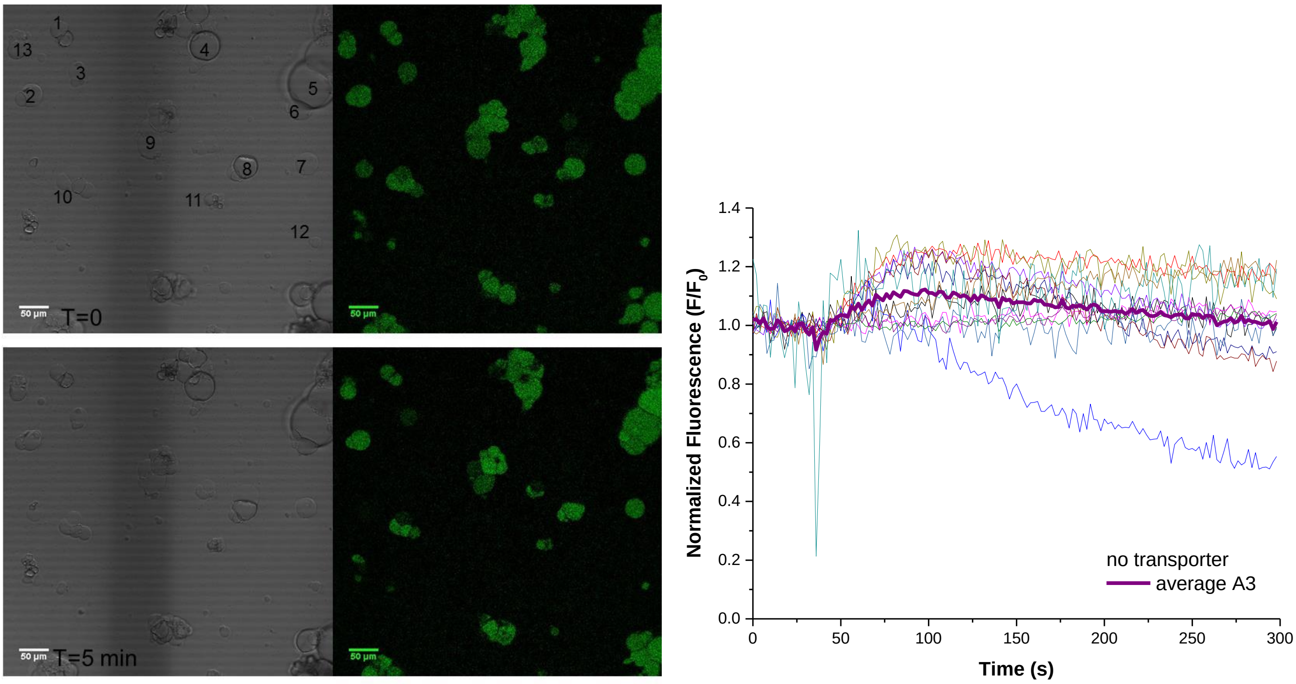

Figure S2 Bright-field and fluorescence microscopy images before (top) and after (bottom) addition of NaCl to GUVs without transporter (*experiment A3*). The normalized fluorescence intensity over time is given for the individual numbered GUVs and for the average of all numbered GUVs (fat purple line). The average diameter of the GUVs used in the average trace for 38 µm.

0.1% transporter

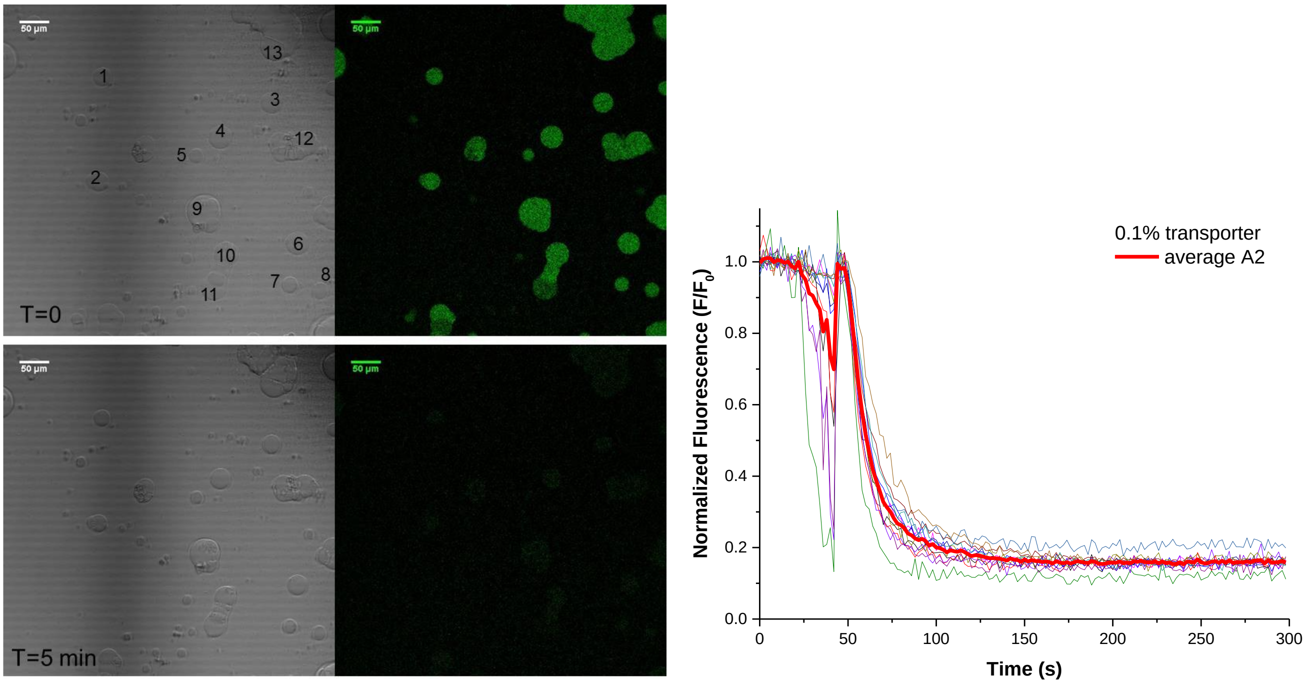

Figure S3 Bright-field and fluorescence microscopy images before (top) and after (bottom) addition of NaCl to GUVs containing 0.1% transporter (*experiment A2*). The normalized fluorescence intensity over time is given for the individual numbered GUVs and for the average of all numbered GUVs (fat red line). The average diameter of the GUVs used in the average trace for 36 µm.[3]

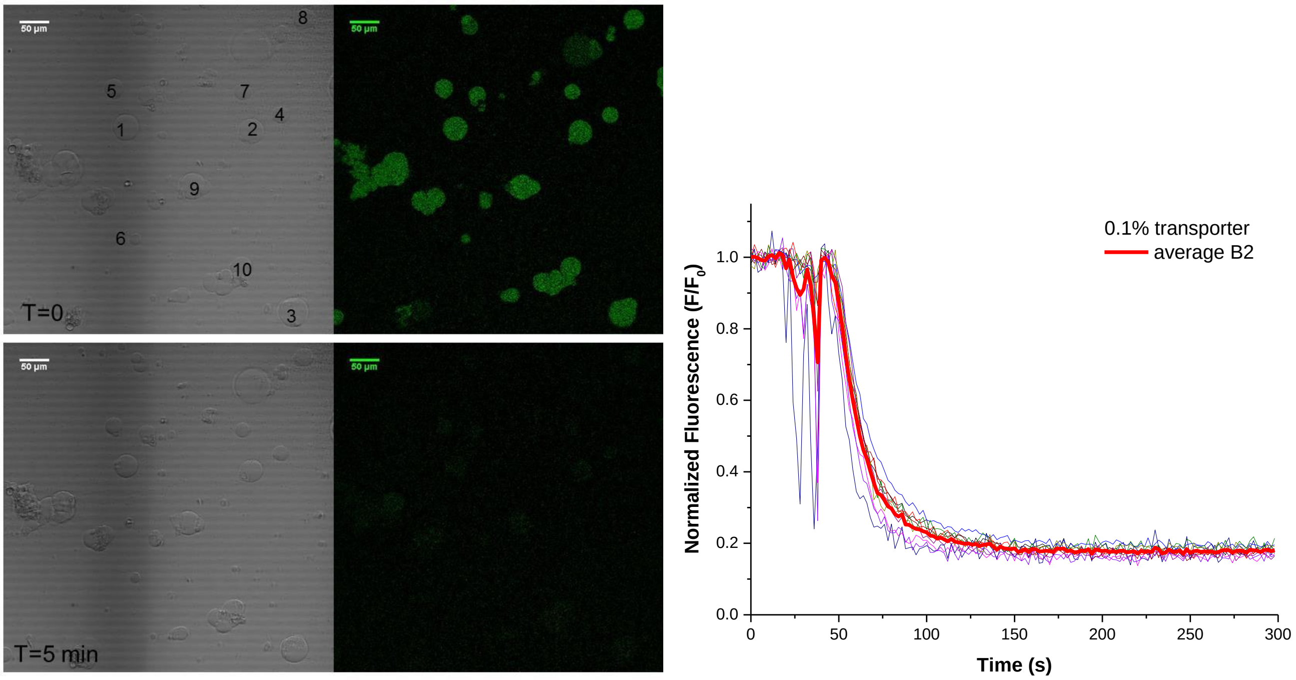

Figure S4 Bright-field and fluorescence microscopy images before (top) and after (bottom) addition of NaCl to GUVs containing 0.1% transporter (*experiment B2*). The normalized fluorescence intensity over time is given for the individual numbered GUVs and for the average of all numbered GUVs (fat red line). The average diameter of the GUVs used for the average trace is 36 µm.

[3] *The decrease of fluorescence intensity before the addition of NaCl (sharp drops before t=0) in some traces is caused by the tip of the syringe used add the NaCl solution passing through the laser beam that is scanning the sample.*

0.04% transporter

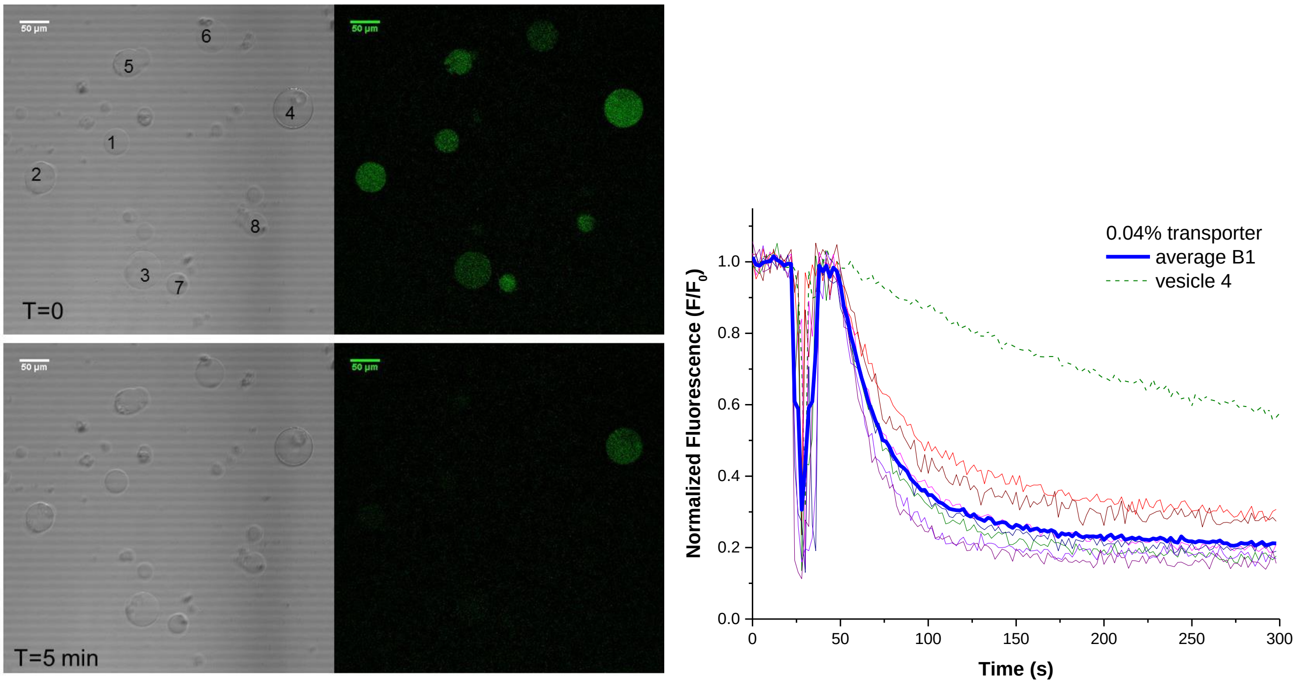

Figure S5 Bright-field and fluorescence microscopy images before (top) and after (bottom) addition of NaCl to GUVs containing 0.04% transporter (*experiment B1*). The normalized fluorescence intensity over time is given for the individual numbered GUVs and for the average of all numbered GUVs (fat blue line; excluded is vesicle 4, corresponding to green dashed line). The average diameter of the GUVs used for the average trace is 46 µm.

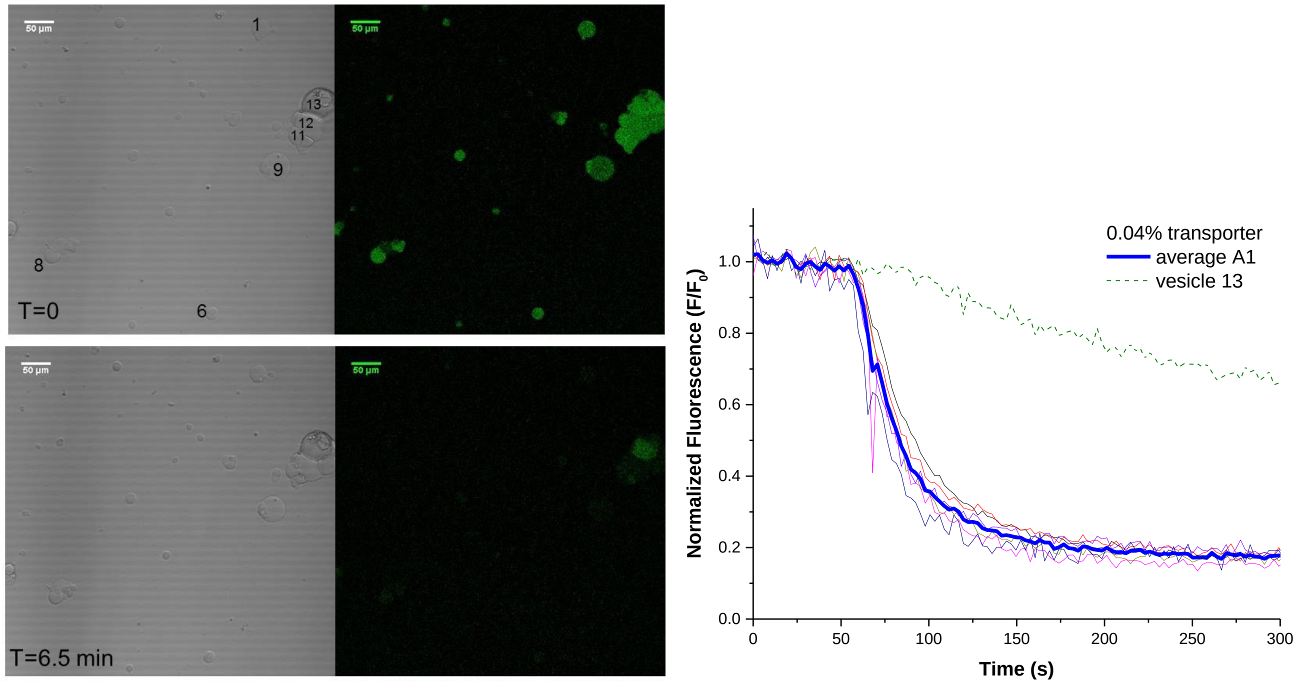

Figure S6 Bright-field and fluorescence microscopy images before (top) and after (bottom) addition of NaCl to GUVs containing 0.04% transporter (*experiment A1*). The normalized fluorescence intensity over time is given for the individual numbered GUVs and for the average of all numbered GUVs (fat blue line; excluded is vesicle 13, corresponding to green dashed line). The average diameter of the GUVs used for the average trace is 34 µm.

0.01% transporter

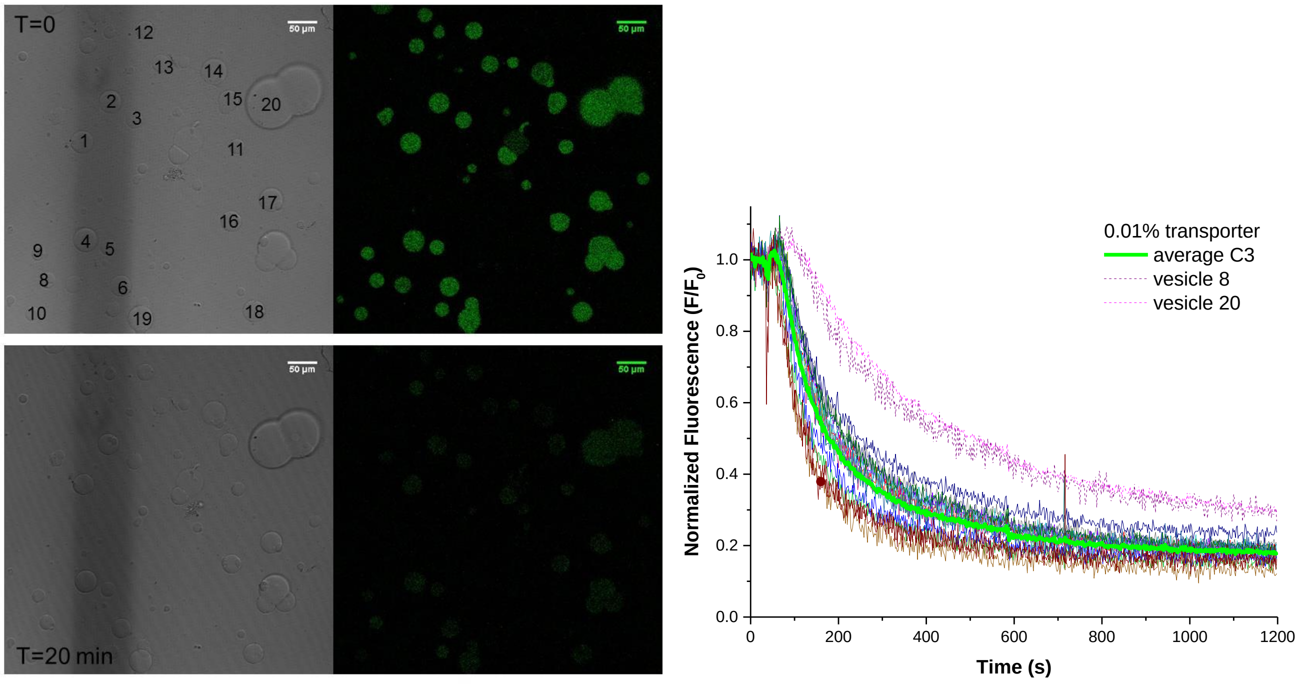

Figure S7 Bright-field and fluorescence microscopy images before (top) and after (bottom) addition of NaCl to GUVs containing 0.01% transporter (*experiment C3*). The normalized fluorescence intensity over time is given for the individual numbered GUVs and for the average of all numbered GUVs (fat light green line; excluded are vesicles 8 and 20). The average diameter of the GUVs used in the average trace for 34 µm.

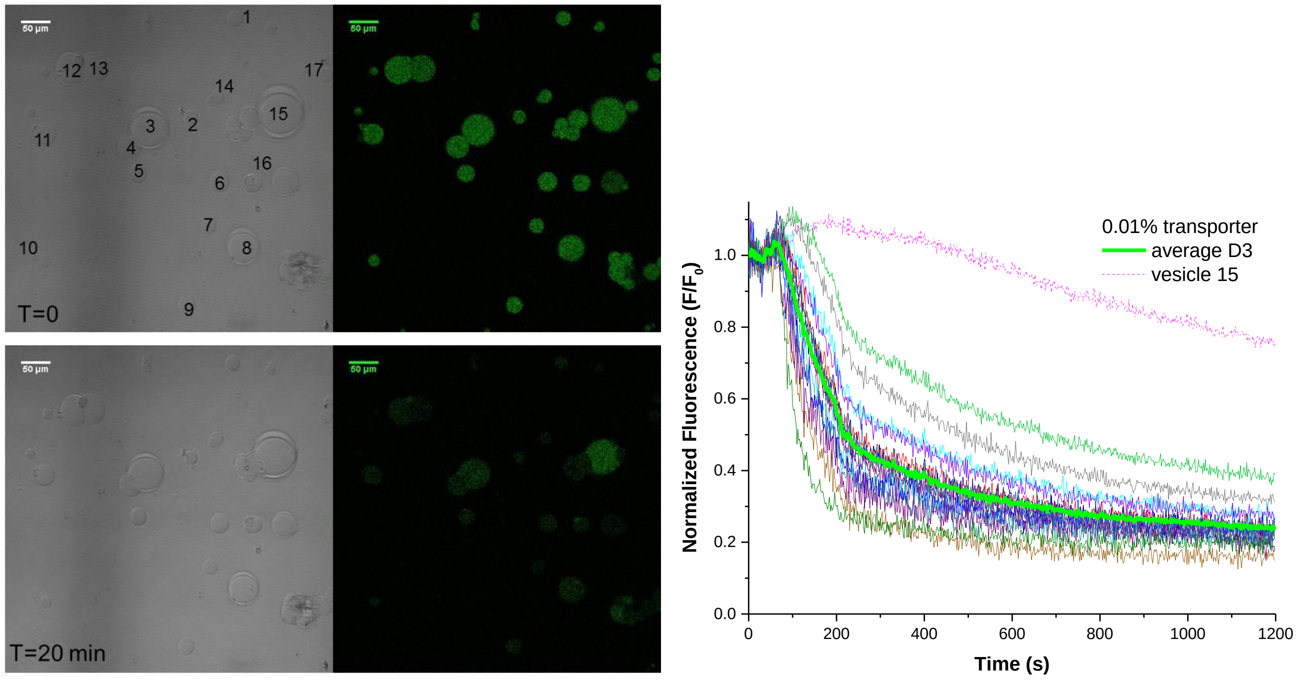

Figure S8 Bright-field and fluorescence microscopy images before (top) and after (bottom) addition of NaCl to GUVs containing 0.01% transporter (*experiment D3*). The normalized fluorescence intensity over time is given for the individual numbered GUVs and for the average of all numbered GUVs (fat light green line; excluded is vesicle 15). The average diameter of the GUVs used in the average trace for 34 µm.

0.01% transporter

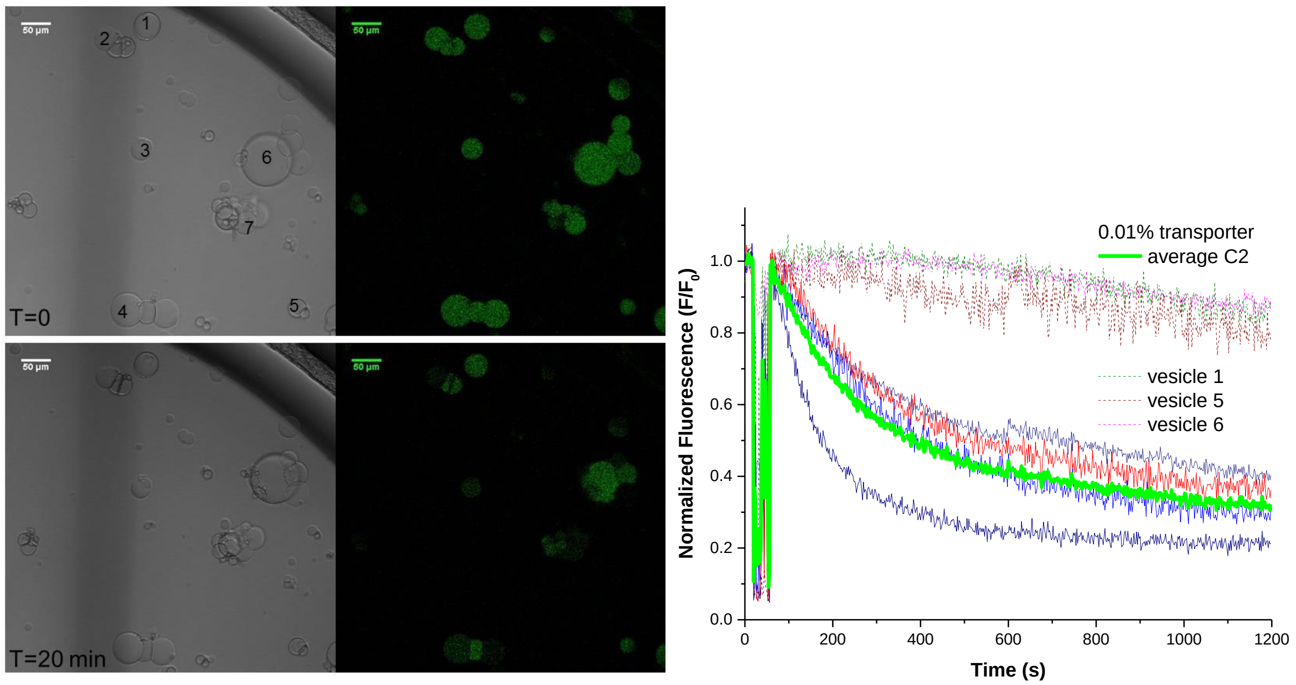

Figure S9 Bright-field and fluorescence microscopy images before (top) and after (bottom) addition of NaCl to GUVs containing 0.01% transporter (*experiment C2*). The normalized fluorescence intensity over time is given for the individual numbered GUVs and for the average of all numbered GUVs (fat light green line; excluded are vesicles 1, 5, and 6). The average diameter of the GUVs used in the average trace for 43 µm.

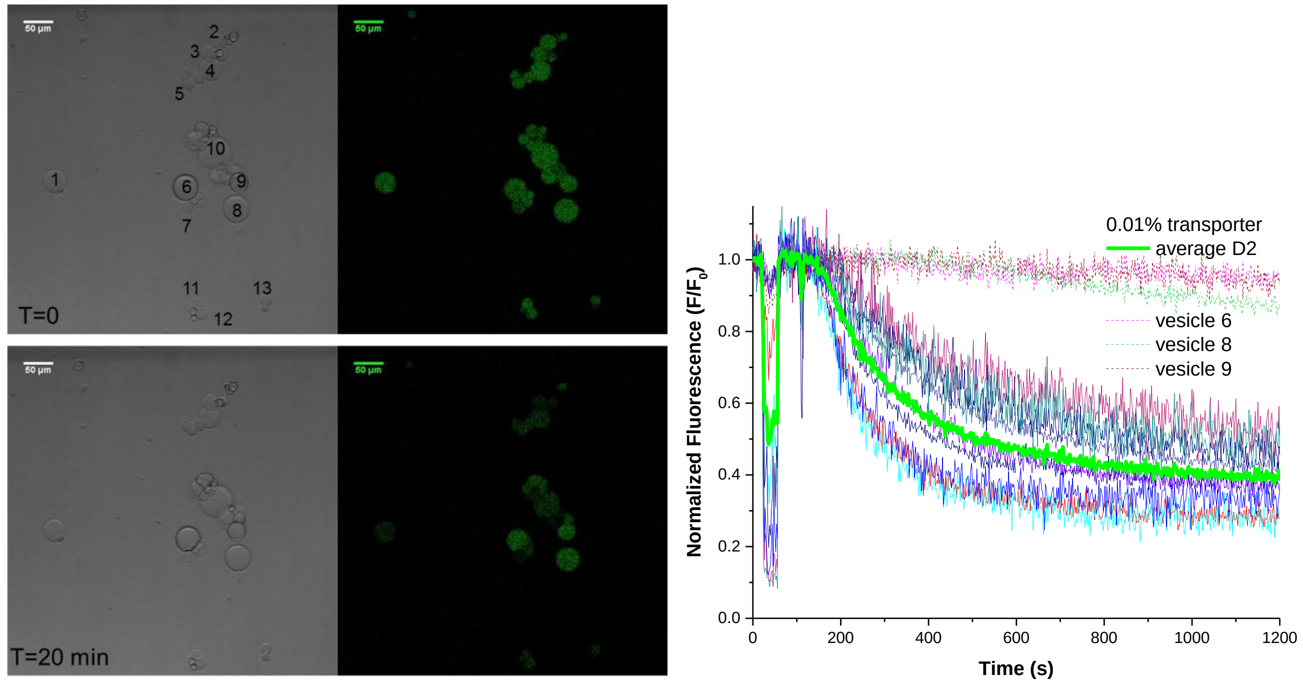

Figure S10 Bright-field and fluorescence microscopy images before (top) and after (bottom) addition of NaCl to GUVs containing 0.01% transporter (*experiment D2*). The normalized fluorescence intensity over time is given for the individual numbered GUVs and for the average of all numbered GUVs (fat light green line; excluded are vesicles 6, 8, and 9). The average diameter of the GUVs used in the average trace for 29 µm.

Test for photobleaching

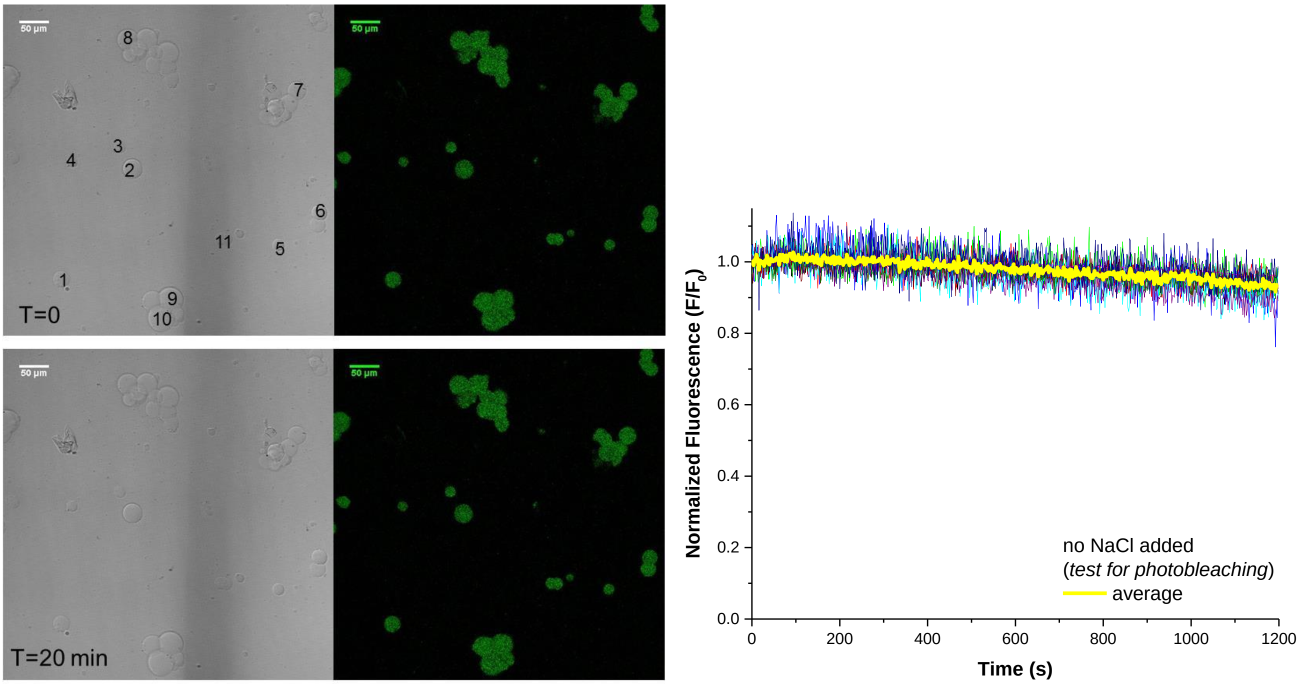

Figure S11 Bright-field and fluorescence microscopy images at the start and end of a 20 minute time lapse measurement to test for photobleaching (no NaCl added). The normalized fluorescence intensity over time is given for the individual numbered GUVs and for the average of all numbered GUVs (fat yellow line).

3. Cross-sections of vesicles with rhodamine-labelled membranes in Figure 4a

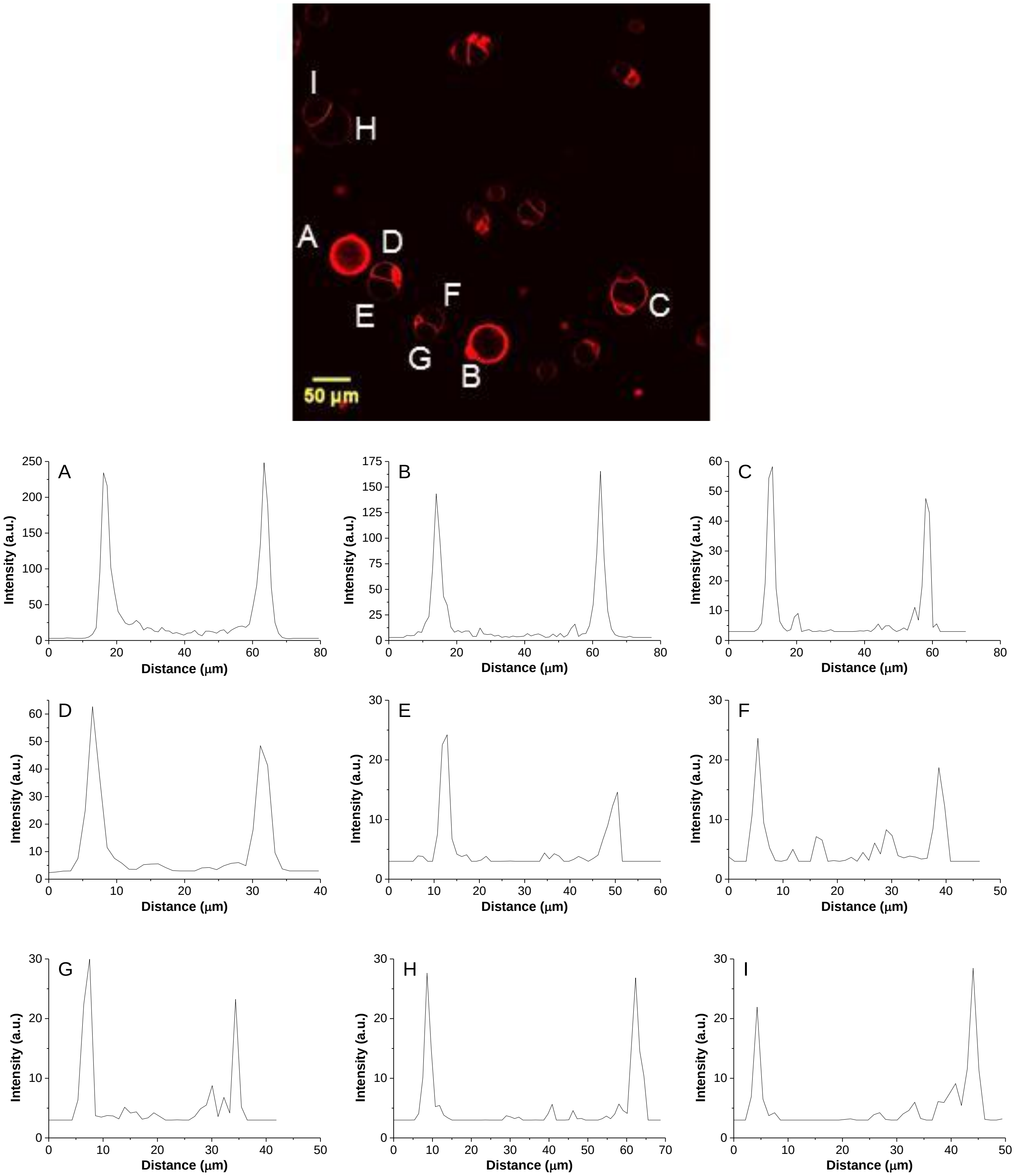

Figure S12 Fluorescence microscopy image of rhodamine labelled lipid **5** in the lipid bilayer membrane (top, copy of Figure 4a of the main paper). Intensity profiles of the cross-sections of the giant vesicles identified with a letter are given, showing intensities ~ 25 for vesicles E-G, ~ 50 for vesicles C and D, ~ 150 for B and ~ 250 for A. From the combination of these data and the transport curves in Figure 4c, we conclude that vesicles E-I are unilamellar and A-D are multilamellar.

4. Fitting of the fluorescence intensities of individual GUVs

According to the Stern-Volmer relationship:

$$\frac{F_0}{F} = 1 + k_q \tau_0 \, [Q]$$

the fluorescence in absence of quencher (F_0) divided by the fluorescence (F) is proportional to the concentration of quencher [Q], which is chloride in our experiments. In the Stern-Volmer equation k_q is the rate constant of the quenching process and τ_0 is the life time of the emitting state of lucigenin, which are both constants. This allows us to take the reciprocal of our normalized fluorescence traces (which are F/F_0) to get F_0/F curves, of which the shape directly represents the concentration of chloride in the vesicles over time.

The first 1000 s of the F_0/F curves obtained for individual GUVs (0.01% transporter) are fitted with a single exponential decay function: $\frac{F_0}{F} = y - a \cdot e^{-kt}$ (1)

Examples of representative fits are given in Figure S13.

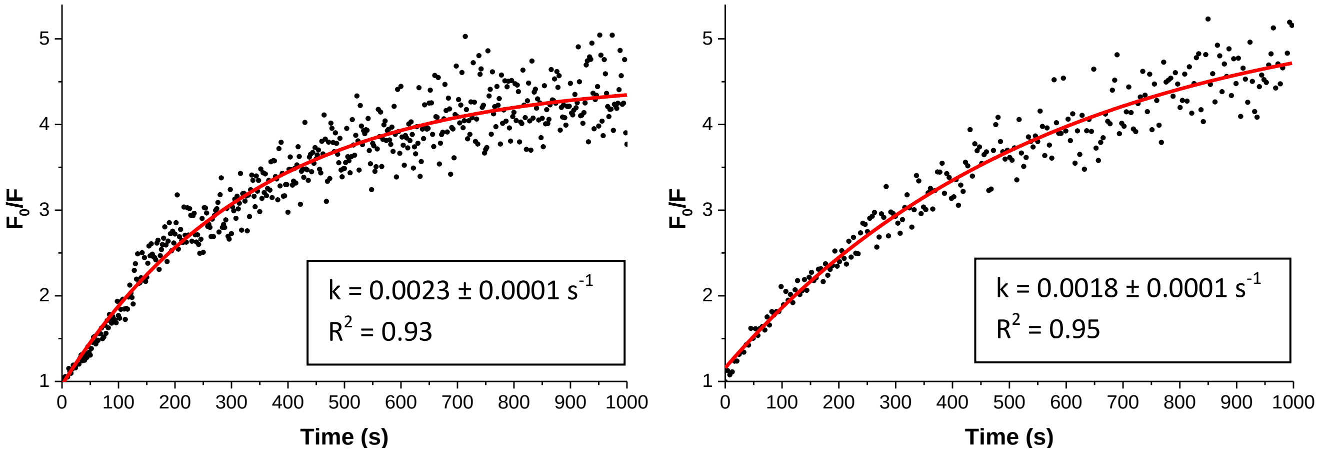

Figure S13 Single exponential fits (red lines) of F_0/F data of individual GUVs (black dots). Note that the noise level of the measured data increases over time. Due to the decreasing fluorescence intensity, the error in measuring the average fluorescence intensity of the area corresponding to a GUV increases.

We note that the increase of the noise in the curves presented in Figure S13 is caused by the decrease of fluorescence intensity upon quenching. The error in measuring the fluorescence intensity thus increases over time when transport is occurring.

The value obtained for parameter k is the rate constant for the transmembrane transport process. Since the fits are generally good we can assume that the concentration of chloride inside the GUV [Cl^-] increases from 0 to 50 mM following the exponential decay function given by equation 2:

$$[Cl^-] = 50\, mM(1 - e^{-kt}) \qquad (2)$$

The rate constant k is obtained from the fit. Differentiation of equation 2 at t=0 gives the initial rate of chloride transport into the GUVs as 50k in mM s^{-1}. From the measured diameter of the GUVs, the volume and the surface area can be calculated. Subsequently, the initial rate (in mM s^{-1}) can be multiplied by the volume of the GUV (V_{GUV}) and Avogadro's number (N_A) to get the initial rate as number of chloride anions transported into the GUV per second. Dividing this by the calculated number of transporters in the GUVs (two times the surface area

A_{GUV}, divided by the surface area per lipid which is assumed to be 46.97 Å^2 for POPC/cholesterol 7:3[4,5] times the ratio of transporter to lipid) gives the initial rate of transport per transporter ($I_{transporter}$) in chloride anions per second (equation 3).

$$I_{transporter} = \frac{50\ mM \cdot k \cdot V_{GUV} \cdot N_A}{\left(\frac{2 \cdot A_{GUV}}{46.97\ \text{Å}^2}\right)\left(\frac{transporter}{lipid}\right)} \qquad (3)$$

Data on 56 GUVs from 6 experiments were analyzed this way, giving an average initial rate per transporter of 820 ± 260 $Cl^- s^{-1}$.

The half-life of the transport process increases linearly with the diameter of the GUVs

The rate of transport by one transporter molecule is independent of the size of the GUV. However, the first-order rate constant for the transport of chloride into the GUVs (k), as obtained from the fits, depends on the volume of the GUV (V_{GUV}) and on the number of transporters present in the membrane and thus on the surface area (A_{GUV}), according to equation (3). From this it can be shown as follows that, for a given transporter at a given concentration, the half-life ($t_{½}$) is directly proportional to the diameter of the GUVs:

As $I_{transporter}$ is a constant, $\frac{k \cdot V_{GUV}}{A_{GUV}}$ must also be constant, indicating that $k \propto \frac{A_{GUV}}{V_{GUV}}$

For half-life ($t_{½}$) one can therefore write:

$$t_{½} = \frac{\ln(2)}{k} \propto \frac{V_{GUV}}{A_{GUV}} \propto \emptyset_{GUV} \text{ (where } \emptyset_{GUV} \text{ is the diameter of the GUVs)}$$

Figure S14a gives a plot of the half-lives obtained for the 16 GUVs from one experiment, which show a nearly linear relationship with diameter of the GUVs. The plot in Figure S14b shows the half-lives from all the GUVs of six experiments (all with 0.01% transporter). The latter plot shows more deviations from the linear relationship between the half-life and the diameter, but the main trend is still linear, indicating how the size of the GUVs affects the observed rate of quenching of the fluorescence.

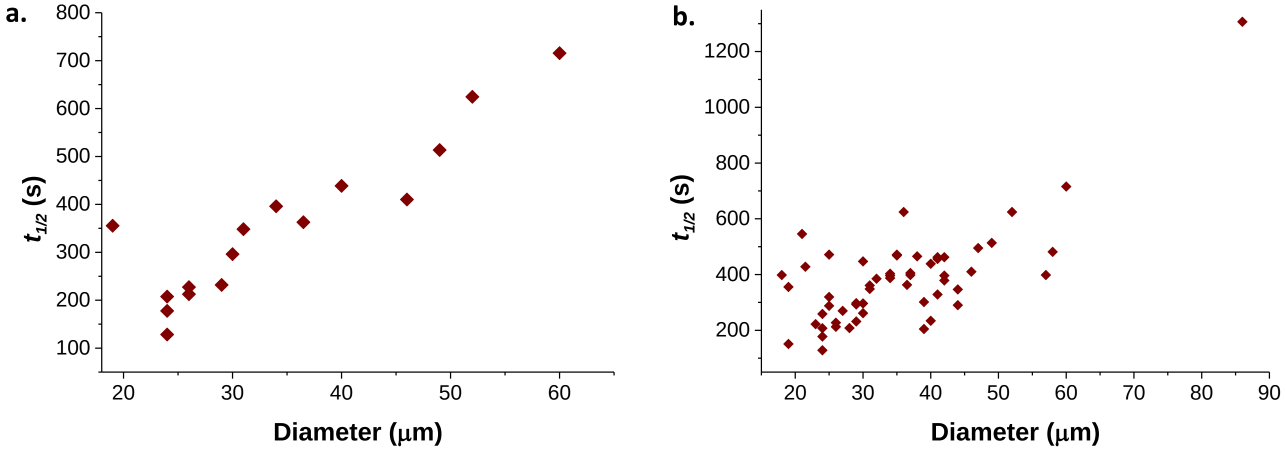

Figure S14 Plot of the half-lives (from single exponential fits, experiments with 0.01% transporter) versus the diameter of 16 individual GUVs from one experiment (a) and from 56 GUVs of 6 experiments combined in one graph (b).

[4] B. N. Olsen, P. H. Schlesinger, N. A. Baker, *J. Am. Chem. Soc.* **2009**, *131*, 4854–4865.
[5] H. Valkenier, L. W. Judd, H. Li, S. Hussain, D. N. Sheppard, A. P. Davis, *J. Am. Chem. Soc.* **2014**, *136*, 12507–12512.

5. Test for leakage of a fluorescent dye from the GUVs

To exclude the possibility that the decrease of fluorescence of the GUVs is caused by lucigenin leaking from the GUVs via defects formed by the transport and chloride, we repeated the experiment with carboxyfluorescein as the fluorescent dye.[6] As the fluorescence of carboxyfluorescein is not quenched by chloride, we should be able to distinguish between the dye leaking from the GUVs and transport of chloride into the GUVs.

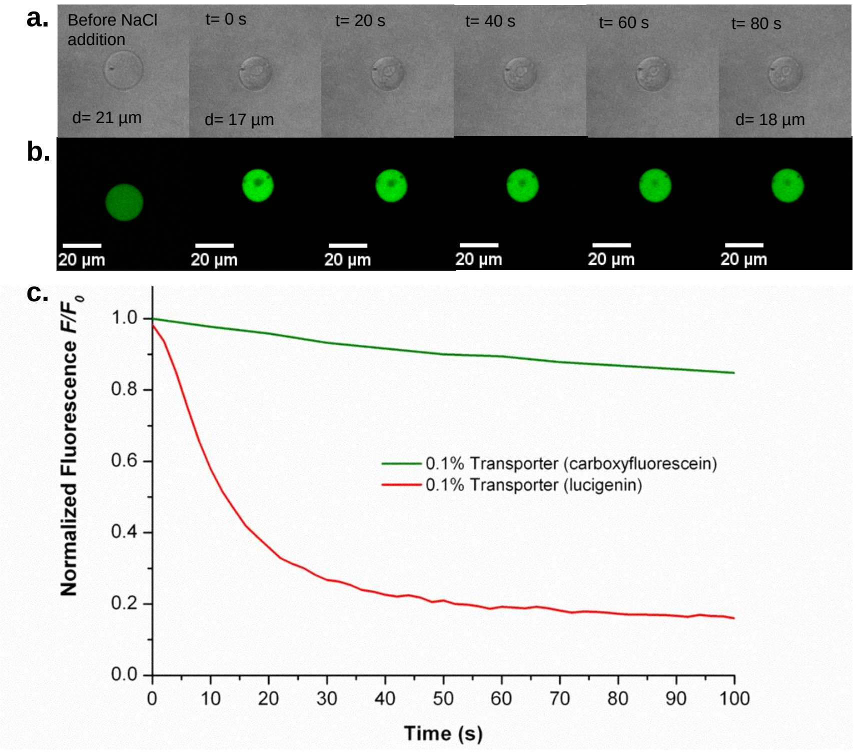

Figure S15 Series of bright-field (**a.**) and fluorescence microscopy images (**b.**) of a GUV made of POPC (70%), cholesterol (30%), and transporter **1** (0.1%) containing carboxyfluorescein (0.1 mM, pH 7) and $NaNO_3$ (225 mM, interior and exterior). The confocal fluorescence imaging is performed with a 488 nm laser at 10% power and a detection filter band between 500-600 nm. Upon the addition of NaCl (25 µL, 1 M) the GUVs shrink producing an increase in the fluorescence, which slightly decreases once the inner content and the exterior reach the equilibrium. **c.** Comparison of the experiments with carboxyfluorescein and lucigenin. The red line corresponds to the average fluorescence of lucigenin with 0.1 % transporter preincorporated in the membrane (see Figure 3 of the main paper). The green line shows the average fluorescence of carboxyfluorescein of 5 individual GUVs with 0.1 % transporter preincorporated in the membrane. The starting time (t = 0 s) corresponds to the addition of 25 µL NaCl (1M).

The results in Figure S15 show that carboxyfluorescein does not leak from the GUVs. We can therefore conclude that the addition of sodium chloride to the GUVs with transporter does not cause the formation of defects or channels in the membrane. This confirms that the observed quenching of fluorescence of lucigenin is caused by chloride transport into the GUVs.

[6] E. E. Ambroggio, F. Separovic, J. H. Bowie, G. D. Fidelio, L. A. Bagatolli, *Biophys J* **2005**, *89*, 1874–1881.
